# *Citrullus colocynthis* as the Cause of Acute Rectorrhagia

**DOI:** 10.1155/2013/652192

**Published:** 2013-05-30

**Authors:** Hamid Reza Javadzadeh, Amir Davoudi, Farnoush Davoudi, Ghasem Valizadegan, Hasan Goodarzi, Sadrollah Mahmoodi, Mohammad Reza Ghane, Mehrdad Faraji

**Affiliations:** ^1^Emergency Medicine Department, Baghiyatallah Hospital, Baghiyatallah University of Medical Sciences, Mulla Sadra, Vanak, Tehran 14758 15943, Iran; ^2^Community and Preventive Medicine Department, Tehran University of Medical Science, Iran; ^3^Gastroenterology Department, Bagheyatallah Medical Student University, Iran

## Abstract

*Introduction*. *Citrullus colocynthis* Schrad. is a commonly used medicinal plant especially as a hypoglycemic agent. *Case Presentation*. Four patients with colocynth intoxication are presented. The main clinical feature was acute rectorrhagia preceeded by mucosal diarrhea with tenesmus, which gradually progressed to bloody diarrhea and overt rectorrhagia within 3 to 4 hours. The only colonoscopic observation was mucosal erosion which was completely resolved in follow-up colonoscopy after 14 days. *Conclusion*. The membranolytic activity of some *C. colocynthis* ingredients is responsible for the intestinal damage. Patients and herbalists should be acquainted with the proper use and side effects of the herb. Clinicians should also be aware of *C. colocynthis* as a probable cause of lower GI bleeding in patients with no other suggestive history, especially diabetics.

## 1. Introduction

*Citrullus colocynthis* Schrad., from the family Cucurbitaceae, popularly named *bitter apple* or *bitter cucumber* in English and called *Hendevaneh Abujahl* (Abujahl watermelon) or *Kadu Hanzal* (bitter ground) in Persian, is a well-known medical plant used alone or in compounds for many medical purposes.

Different parts of the plant including seeds, fruit, root, stem, and leaves, used as either aqueous or oil extracts, dried or fresh, are believed to have antidiabetic [1–6], antihyperlipidemic [7, 8], laxative [1, 3, 9], anti-inflammatory [9], analgesic [9], vermifuge [5], hair-growth-promoting [10], antibacterial [11], antifungal [11], and antioxidant properties [12].

In spite of multiple medical benefits, some of the most frequently reported complications such as colic, diarrhea, hematochezia, nephrosis, vomiting, and liver impairment [4, 13, 14] have placed *C. colocynthis* amongst the top 10 toxic plants [14].

In 1989, three cases of toxic acute colitis following ingestion of *C. colocynthis* were reported by Goldfain et al. The main clinical presentation was dysenteric diarrhea. The colonoscopic observations were mucosal congestion and hyperemia with abundant exudates, but no ulceration or pseudopolyp formation, which disappeared within 14 days in all patients [15]. Contreras et al. also reported colocynth poisoning as a rare cause of acute diarrhea syndrome in 1996 [16]. Khan et al. reported 5 cases of toxicity due to consumption of colocynth in Saudi Arabia over a 2-year period who presented with acute severe bloody diarrhea [17].

## 2. Case Presentation

### 2.1. Patient I

A 28-year-old white housewife, with body mass index of 24 kg/m^2^, presented to the emergency department (ED) with rectorrhagia from 2 hours prior to admission. She had been experiencing a mucosal diarrhea with tenesmus, which gradually progressed to bloody diarrhea and overt rectorrhagia within 4 hours. 

The history of common perianal causes of rectorrhagia (hemorrhoid, fissure), inflammatory bowel disease (IBD), and peptic ulcer disease (PUD) was negative. The patient suffered from intermittent episodes of constipation for which she mainly used olive oil and took about 1.5 g of *Citrullus* dried fruit for the first time on the day of admission. The patient was on no medications.

### 2.2. Patient II

A 32-year-old white housewife, with BMI of 26 kg/m^2^, came to ED complaining from rectorrhagia one hour prior to admission, reporting a sudden onset of mucosal diarrhea and tenesmus followed by bloody diarrhea and eventually ended to rectorrhagia within 3 hours. The patient had no history of hemorrhoid, fissures, IBD, or PUD. She was diagnosed with type II diabetes 6 months earlier. She took no medications and started using *Citrullus* fresh fruit twice a day (1.6 g/d)—as a herbal hypoglycemic agent—for the previous two days.

### 2.3. Patient III

 A 57-year-old white housewife, with BMI of 32 kg/m^2^, was admitted to the ED with rectorrhagia from 3 hours earlier. She had a burst of mucosal diarrhea with tenesmus, which gradually progressed to bloody diarrhea and overt rectorrhagia within 3 hours. She mentioned no perianal causes of rectorrhagia, no history of IBD or PUD. The patient has been a known case of type II diabetes for 8 years. Her fasting plasma glucose was poorly controlled in spite of taking 500 mg metformin daily. She started to use 2 cups of brewed extract of *Citrullus* as a herbal hypoglycemic agent 4 days before. 

The patient experienced a right ventricular rapid response atrial fibrillation 1 hour after admission which was controlled after rehydration with 1100 cc of normal saline intravenous infusion. 

### 2.4. Patient IV

A 45-year-old white housewife, with BMI of 28 kg/m^2^, presented to the ED with rectorrhagia for the previous 2 hours, preceded by mucosal diarrhea and tenesmus which progressed to bloody diarrhea in 3.5 hours. She had no history of hemorrhoid, fissures, IBD, or PUD and was diagnosed with type II diabetes 3 months earlier. The patient was on no medications and she took 2 cups of brewed extract of *Citrullus* as a herbal hypoglycemic agent for the past two days.

All patients were hemodynamically stable. The physical examination of all four patients revealed no sign of perianal fissures or hemorrhoid and no indications for emergency abdominal surgery. The nasogastric tubes expelled clear gastric secretions. The stool examination further ruled the infectious causes out. The upper gastrointestinal (GI) endoscopy showed no upper GI source of bleeding. In the colonoscopic examination, none of the patients had ulcerations, polyp formations, or any other abnormality but mucosal erosion.  Table 1 summarizes main clinical and paraclinical features of the patients.

**Figure 1:**
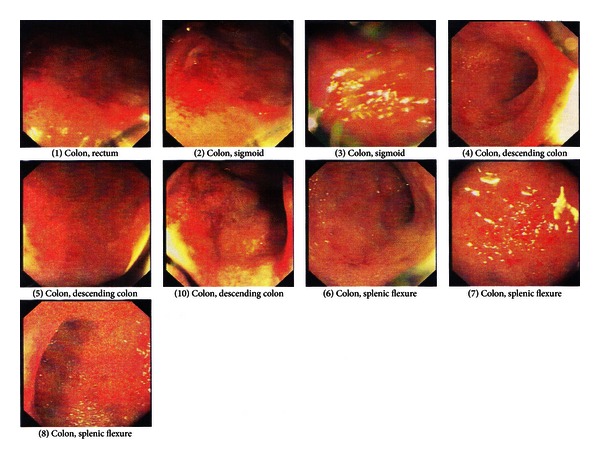
Sample colonoscopic findings at admission.

Proper IV line, normal saline infusion (to keep veins open), and pantoprazole were ordered for all patients. Patients I and II received one dose of metronidazole according to the consultations made with infectious disease specialist, but discontinued the treatment as further investigations ruled dysentery out of the differential diagnosis list (Figure 1).

Patients I and II were discharged from the ED observation room to home after 36 and 42 hours, respectively. With the experience of the first two patients in mind, we specifically asked patients III and IV about herbal medications including *C. colocynthis, *which helped to shorten their hospitalization duration to 18 and 16.5 hours.

The follow-up colonoscopy after 14 days was completely normal with no sign of erosions.

## 3. Conclusion

*C. colocynthis *has been demonstrated to be responsible for diarrhea in treated animals [18] and colitis in humans [14–17]. The membranolytic activity of saponin—an ingredient of the pulp extract—is believed to be the main pathophysiological mechanism for intestinal damage [6].

The acceptable dosage of *C. colocynthis* fruit ranges from 0.6 to 1.75 g/day and 0.1–0.4 g/day, according to Traditional Iranian Medicine (TIM) and modern phytotherapy, respectively. The seed should be administered at 120–300 mg (max: 600 mg) per day, and the root powder from 0.2 to 0.4 g/day. If the fruit is administered with its correctives such as Arabic gum, the adverse events are reduced and larger doses are allowed [5].

All the four presented patients had ingested considerably larger amounts of *C. colocynthis* than the permitted dose. Additionally, they took the whole fruit (saponin-containing pulps) and used no correctives which in turn have been the augmentations to intoxication.

The *C. colocynthis *overdose seems to be the reasonable cause of the mentioned clinical scenarios due to the biological plausibility, temporal relationship, analogy (i.e., consideration of alternate explanations), and coherence.

There are no approved products of *C. colocynthis *available in pharmacies or herbal drugstores. In the absences of official surveillance and proper patient education, the herb could frequently be administered and taken inappropriately. 

We strongly suggest acquainting herbalists with side effects, allowed and toxic dosage of different parts of the fruit, encouraging use of seeds (containing no saponin) instead of the whole fruit or pulps, and considering recommendations of TIM such as using *C. colocynthis *correctives or grinding the herb.

Clinicians should also be aware of *C. colocynthis* as a probable cause of lower GI bleeding in patients with no other suggestive history, especially diabetics, and carefully ask about it. Additionally physicians can actively take part in educating people about dosage and side effects of herbal medicine frequently used by their patients.

## Figures and Tables

**Table 1:** Summary of patients' characteristics and findings.

	Age	Sex	Ingested form	Therapeutic purpose	Presentation	Course	Clinical findings	Lab findings	Imaging findings	Colonoscopic findings
I	28 y	F	Dried pulp	Laxative agent	Rectorrhagia	Diarrhea progressing to bloody diarrhea and overt rectorrhagia in 4 hours	Tenesmus (+) Nausea (−), vomiting (−) Abdominal tenderness/rebound tenderness (−) BP = 110/70 mmHg (tilt sign (−)) PR = 70/min, RR = 14/min, *T* = 37^c^ O_2_ saturation = 96%	HB = 12.5 ∗∗/∗∗ ↓ in 6-hours followup: (−)	X-Ray: NL US: NL	Mucosal erosion

II	32 y	F	Fresh fruit	Hypoglycemic agent	Rectorrhagia	Diarrhea progressing to bloody diarrhea and overt rectorrhagia in 3 hours	Tenesmus (+) Nausea (−), vomiting (−) Abdominal tenderness/rebound tenderness (−) BP = 130/70 mmHg (tilt sign (−)) PR = 77/min, RR = 13/min, *T* = 37.2^c ^ O_2_ saturation = 96%	HB = 14 ∗∗/∗∗ ↓ in 6-hours followup: (−)	X-Ray: NL US: NL	Mucosal erosion

III	57 y	F	Brewed extract	Hypoglycemic agent	Rectorrhagia	Diarrhea progressing to bloody diarrhea and overt rectorrhagia in 3 hours	Tenesmus (+) Nausea (−), vomiting (−) Abdominal tenderness/rebound tenderness (−) BP = 130/70 mmHg (tilt sign (−)) PR = 81/min, RR = 14/min, *T* = 37.2^c ^ O_2_ saturation = 94%	HB = 12.5 ∗∗/∗∗ ↓ in 6-hours followup: (−)	X-Ray: NL US: NL	Mucosal erosion
						The patient experienced an AF RVR 1 hour after admission which was controlled with supportive treatments	During the course of AF: BP = 105/95 mmHg PR > 100/min, RR = 16/min O_2_ saturation = 96%	Na = 131 K = 3.5		

IV	45 y	F	Brewed extract	Hypoglycemic agent	Rectorrhagia	Diarrhea progressing to bloody diarrhea and overt rectorrhagia in 3.5 hours	Tenesmus (+) Nausea (−), vomiting (−) Abdominal tenderness/rebound tenderness (−) BP = 110/95 mmHg (tilt sign (−)) PR = 81/min, RR = 13/min, *T* = 37.2^c ^ O_2_ saturation = 95%	HB = 13.7 ∗∗/∗∗ ↓ in 6-hours followup: (−)	X-Ray: NL US: NL	Mucosal erosion

## References

[1] Abdel-Hassan IA, Abdel-Barry JA, Mohammeda ST (2000). The hypoglycaemic and antihyperglycaemic effect of Citrullus colocynthis fruit aqueous extract in normal and alloxan diabetic rabbits. *Journal of Ethnopharmacology*.

[2] Ardekani MRS, Rahimi R, Javadi B, Abdi L, Khanavi M (2011). Relationship between temperaments of medicinal plants and their major chemical compounds. *Journal of Traditional Chinese Medicine*.

[3] Huseini HF, Darvishzadeh F, Heshmat R, Jafariazar Z, Raza M, Larijani B (2009). The clinical investigation of Citrullus colocynthis (L.) schrad fruit in treatment of type II diabetic patients: a randomized, double blind, placebo-controlled clinical trial. *Phytotherapy Research*.

[4] Nmila R, Gross R, Rchid H (2000). Insulinotropic effect of Citrullus colocynthis fruit extracts. *Planta Medica*.

[5] Rahimi R, Amin G, Ardekani MRS (2012). A Review on Citrullus colocynthis Schrad.:from traditional Iranian medicine to modern phytotherapy. *Journal of Alternative and Complementary Medicine*.

[6] Shafaei H, Esmaeili A, Rad HS, Delazar A, Behjati M (2012). Citrullus colocynthis as a medicinal or poisonous plant: a revised fact. *JMPR*.

[7] Daradka H, Almasad MM, Qazan WS, El-Banna NM, Samara OH (2007). Hypolipidaemic effects of Citrullus colocynthis L. in rabbits. *Pakistan Journal of Biological Sciences*.

[8] Rahbar AR, Nabipour I (2010). The hypolipidemic effect of citrullus colocynthis on patients with hyperlipidemia. *Pakistan Journal of Biological Sciences*.

[9] Marzouk B, Marzouk Z, Haloui E, Fenina N, Bouraoui A, Aouni M (2010). Screening of analgesic and anti-inflammatory activities of Citrullus colocynthis from southern Tunisia. *Journal of Ethnopharmacology*.

[10] Dhanotia R, Chauhan NS, Saraf DK, Dixit VK (2009). Effect of Citrullus colocynthis Schrad fruits on testosterone-induced alopecia. *Natural Product Research*.

[11] Marzouk B, Marzouk Z, Décor R (2009). Antibacterial and anticandidal screening of Tunisian Citrullus colocynthis Schrad. from Medenine. *Journal of Ethnopharmacology*.

[12] Tannin-Spitz T, Bergman M, Grossman S (2007). Cucurbitacin glucosides: antioxidant and free-radical scavenging activities. *Biochemical and Biophysical Research Communications*.

[13] Dehghani F, Panjehshahin MR (2006). The toxic effect of alcoholic extract of Citrullus colocynthis on rat liver. *Iranian Journal of Pharmacology and Therapeutics*.

[14] Jouad H, Haloui M, Rhiouani H, El Hilaly J, Eddouks M (2001). Ethnobotanical survey of medicinal plants used for the treatment of diabetes, cardiac and renal diseases in the North centre region of Morocco (Fez-Boulemane). *Journal of Ethnopharmacology*.

[15] Goldfain D, Lavergne A, Galian A, Chauveinc L, Prudhomme F (1989). Peculiar acute toxic colitis after ingestion of colocynth: a clinicopathological study of three cases. *Gut*.

[16] Contreras MCG, Gallardo AL, Garcia FD, Rodriguez FY (1996). Poisoning by coloquintide, an infrequent cause of acute diarrhoea syndrome. *Medicina Clinica*.

[17] Khan SA, Shelleh HH, Bhat AR, Bhat KS (2003). Colocynth toxicity. A possible cause of bloody diarrhea. *Saudi Medical Journal*.

[18] Al Faraj S (1995). Haemorrhagic colitis induced by Citrullus colocynthis. *Annals of Tropical Medicine and Parasitology*.

